# Characterization of a Human 12/15-Lipoxygenase Promoter Variant Associated with Atherosclerosis Identifies Vimentin as a Promoter Binding Protein

**DOI:** 10.1371/journal.pone.0042417

**Published:** 2012-08-07

**Authors:** Susmita Samanta, Kurtis Anderson, Sean Moran, David Hawke, David Gorenstein, Myriam Fornage

**Affiliations:** 1 Research Center for Human Genetics, Brown Foundation Institute of Molecular Medicine, University of Texas Health Science Center at Houston, Houston, Texas, United States of America; 2 Centers for Proteomics and Systems Biology, Brown Foundation Institute of Molecular Medicine, University of Texas Health Science Center at Houston, Houston, Texas, United States of America; 3 Human Genetics Center, School of Public Health, University of Texas Health Science Center at Houston, Houston, Texas, United States of America; 4 Department of Biochemistry and Molecular Biology, University of Texas Medical Branch, Galveston, Texas, United States of America; 5 Department of Biochemistry & Cell Biology, Rice University, Houston, Texas, United States of America; 6 Department of Molecular Pathology, M.D. Anderson Cancer Center, Houston, Texas, United States of America; University Medical Center Utrecht, The Netherlands

## Abstract

**Background:**

Sequence variation in the human 12/15 lipoxygenase (ALOX15) has been associated with atherosclerotic disease. We functionally characterized an ALOX15 promoter polymorphism, rs2255888, previously associated with carotid plaque burden.

**Methodology/Principal Findings:**

We demonstrate specific *in vitro* and *in vivo* binding of the cytoskeletal protein, vimentin, to the ALOX15 promoter. We show that the two promoter haplotypes carrying alternate alleles at rs2255888 exhibit significant differences in promoter activity by luciferase reporter assay in two cell lines. Differences in i*n-vitro* vimentin-binding to and formation of DNA secondary structures in the polymorphic promoter sequence are also detected by electrophoretic mobility shift assay and biophysical analysis, respectively. We show regulation of ALOX15 protein by vimentin.

**Conclusions/Significance:**

This study suggests that vimentin binds the ALOX15 promoter and regulates its promoter activity and protein expression. Sequence variation that results in changes in DNA conformation and vimentin binding to the promoter may be relevant to ALOX15 gene regulation.

## Introduction

Foam cell formation and oxidative modification of low density lipoprotein (LDL) are some of the mechanisms of atherosclerosis pathogenesis [1]. Human arachidonate 15-lipoxygenase, a non-heme iron dioxygenase highly expressed in macrophages, has been shown to participate in these processes [2], [3], [4], [5], [6], but its precise role in atherosclerosis is controversial. Both higher and lower activity of the enzyme has been reported to be associated with atherosclerosis in different experimental models [7], [8]. Sequence variations in the promoter of the reticulocyte-type 15-lipoxygenase gene (ALOX15) has been associated with cardiovascular disease [5], [8], [9]. The G allele of a single nucleotide polymorphism (SNP) rs2255888 in the human ALOX15 promoter region has been associated with carotid plaque burden [10]. However, detailed studies are needed to investigate the role of such variant in ALOX15 regulation and function, and its relevance to disease.

Vimentin, a cytoskeletal protein, is strongly expressed in foam cells [11], smooth muscle cells of human atheromatous plaques [12], and in differentiated human monocytes [13], [14]. Increased expression of vimentin has been shown in monocytes from coronary artery disease patients [15], in THP-1 human monocytes stimulated by oxidized LDL [16], and in growth factor-exposed aortic vascular smooth muscle cells (VSMC) [17]. Vimentin has also been shown to be secreted by activated macrophages [18] and endothelial cells [19]. Presence of vimentin in nuclear extract preparations as well as DNA-mediated import of vimentin into the nucleus have been previously reported [20]. In addition to its known role as a cytoskeletal protein in maintaining cell shape, multifunctional roles have been ascribed to vimentin, including roles in the organization and regulation of proteins involved in cell adhesion, migration, and stress-mediated cell signaling [21], [22], [23]. Moreover, vimentin has been shown to interact through its N-terminal non-α-helical head domain, with G-rich highly repetitive DNA sequences [24]. Structural similarities with transcription factors along with the possible involvement of vimentin in different DNA-dependent nuclear events have also been noted [25], [26].

Here we report specific binding of vimentin to the region of the ALOX15 promoter encompassing rs2255888. We show differential vimentin-mediated promoter activity between haplotype sequences carrying either the G or A allele at rs2255888. We also demonstrate a difference in DNA structure and vimentin-binding ability of the two promoter haplotypes.

## Materials and Methods

### Reagents and Buffers

Reagents are shown in Materials S1.

### Cloning of the ALOX15 Promoters and cDNA

**Figure 1:**
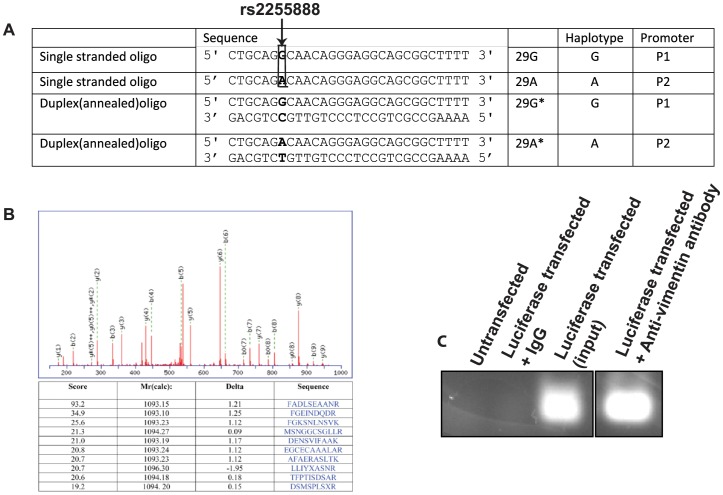
In vitro and In vivo Binding of Vimentin to Human ALOX15 Promoter Variants. (**A**) Oligonucleotide sequences used for the gel shift assay experiments corresponding to promoters P1 and P2, which differ at rs2255888 (indicated by arrow). (**B**) Identification and analysis of Vimentin, from magnetic bead DNA-protein pull-down assay, by LC-MS/MS. Proteins were identified by database search of the fragment spectra against the National Center for Biotechnology Information nonredundant protein database (NCBInr) using Mascot (version 2.2, Matrix Science, London, UK). The sequence match for one of the assigned peptides is shown with the fragment ions identified as y(n), b(m) to indicate the y and b ions, respectively. The table shows the top-ranked peptide match and the scores for the next 9 for that spectrum. (**C**) Chromatin immunoprecipitation (ChIP) assay of P1 luciferase transfected NIH3T3 cells with anti-vimentin antibody (C20).

A 594-bp promoter sequence of the ALOX15 gene of (−701 to −108 upstream from the translational start codon) was PCR amplified with appropriate primers (Fig. 1A). The PCR-amplified ALOX15 promoter fragment was cloned into a pGL4.10 (luc2) vector. Two constructs carrying either the G- or A-haplotype sequences were made. ALOX15 was cloned in pCMV-Tag2 using forward and reverse primer having BamH1 and Xho1 sequence, respectively (see Materials S1 for details).

### Transient Transfection

Transient transfection of NIH3T3 cells with cloned luciferase constructs (P1 and P2) were carried out with Fugene 6 (Roche Applied Science, Indianapolis, IN) according to manufacturer’s protocol. NIH3T3 cells (3.5×10^4^) and MCF-7 cells (0.5×10^4^) were seeded in 24-well plates and 96-well plates, respectively. Transfection was carried out in NIH3T3 cell (a (mouse fibroblast cell line) with 200 ng of luciferase constructs. MCF-7 was transfected with luciferase constructs with or without ALOX15 cDNA. Transient transfection of BPH-1 cell, a benign prostate cell line, was carried out with vimentin cDNA (Origene, Rockville, MD) and vector (CMV) DNA in 6-well plate (2.4×10^5^ cells/well) with Fugene 6 following the manufacturer’s protocol.

### Luciferase Activity

Luciferase activity was measured after 24 hours of transfection in NIH3T3 cells and MCF-7 cells using a dual luciferase kit from Promega (Promega Corporation, Madison,WI). The experiments were done in triplicates. The results are the average of three independent transfections each of which performed in triplicate ± S.E. Luciferase activity was measured according to the manufacturer’s protocol and was normalized using Renilla luciferase activity for NIH3T3 cells and TK-luciferase activity for MCF-7 cells in each sample.

### DNA Pull Down Assay

Washed streptavidin beads were incubated with biotinylated 29G* annealed oligos (20 pM), washed and used for mass spectrometry using LC/MS/MS mass spectrometer to identify the bound proteins (see Materials S1 for details).

### Chromatin Immunoprecipitation (CHIP) Assay

Nuclear proteins were cross-linked to Pl luciferase construct using CHIP assay kit (Active Motif, Carlsbad, CA) and then magnetic beads coupled with protein G and anti-vimentin antibody C-20 (1 µg) were used to capture the chromatin-immunoprecipitate. Five microliter of eluted DNA from DNA-protein complex was used for PCR amplification with promoter-specific primers. The PCR product was subjected to electrophoresis on 1.5% agarose gels, stained with ethidium bromide (see Materials S1 for details).

### Nuclear Extracts Preparation

Cultured cells were washed with ice-cold phosphate-buffered saline (PBS) and recovered in 1 ml of PBS. Nuclear extract was prepared using NE-PER nuclear and cytoplasmic extraction reagent (Thermo Scientific, Rockford, IL) according to manufacturer’s protocol. The nuclear extract contains 40 mM salt. Halt protease and phosphatase inhibitors were used during cytoplasmic and nuclear extraction.

### Electrophoretic Mobility Shift Assay (EMSA)

The HPLC purified biotinylated oligos were annealed with their respective complementary strands to make duplex oligos in an annealing buffer. NIH3T3 nuclear extracts (10 µg) was incubated with biotinylated oligos in a buffer (20 mM Tris-HCl pH 8.0, 0.4 mM EDTA, 0.4 mM DTT, 5% glycerol) and in the presence of 1% NP40, poly dI/dC at 4°C for 40 min respectively. Then 6% non-denaturing polyacrylamide was run, blotted and developed using chemiluminescent nucleic acid detection module (supplemental Materials S1).

### Denaturing Polyacrylamide Gel Electrophoresis and Western Blotting

BPH cells were transfected with vimentin cDNA and CMV vector cDNA. After 48 h the cells were harvested and cell lysates were run on a 4–20% precast polyacrylamide gel (Bio-Rad, Hercules, CA). The blot was probed with anti-ALOX15 antibody (1∶2000), anti vimentin antibody (1∶1000) and anti-β-actin antibody (1∶7000) followed by corresponding secondary antibodies.

### Nondenaturing Polyacrylamide Gel Electrophoresis

Duplex oligos were incubated in the same binding buffer that was used for EMSA and 15% polyacrylamide gel electrophoresis was performed in 1X TBE buffer containing 40 mM NaCl at 4°C. After pre-running the gel for 30 min the samples were loaded and run in the cold. The gel was blotted and bands were detected as earlier described [27].

### Ultraviolet Thermal Melting Studies (UVM)

Purity of the oligo was estimated at >95% using denaturing polyacrylamide gel electrophoresis with 1 µg sample loaded. Single-stranded oligonucleotides were extensively dialyzed into thermal transition buffer containing 3.87 mM NaH2PO4; 6.13 mM Na2H2PO4; and 23.86 mM NaCl to equal a final [Na+] of 40 mM, adjusted to pH 7.0+/0.05 with NaOH. Thermal transition experiments were carried out on a Cary 1 UV-Vis with an attached thermostatable Peltier accessory inside a 1 cm path-length sealed quartz cuvette. Concentration of single-stranded oligomers were determined from absorbance at 260 nm using estimated extinction coefficients and mass measurements of the sample showed no evidence of evaporation (<0.01% w). Absorbance at 295 nm was used to monitor the unfolding transition as described previously for G-quadruplex structures [28]. Absorbance values, A295, were measured every 0.1°C in the temperature range of 15–80°C, starting with an initial room temperature A295 value of 1.0 for both samples. Both heating (unfolding) and cooling (refolding) transition curves were recorded at a constant rate of temperature change of 0.4°C/ min monitored from an internal probe located inside of the Peltier holder (see Materials S1).

### Circular Dichroism (CD)

The single stranded oligos were dialyzed overnight in a buffer containing 40 mM Na+, 10 mM PO4, pH 7.0 in a total volume of 300 µl. The spectral analysis was performed on a by Jasco 715 spectrophotometer using ac cell with a path length of 0.1 mm.

### Absorption Spectra

All measurements were performed on a Cary 1 E UV/Vis spectrophotometer using quartz cuvettes with an optical path length of 1 cm. Absorption spectra were recorded in 220–320 nm range. Thermal differential spectra were plotted by dividing the difference of absorbance at 90°C and 20°C at particular wavelength by its highest Δ absorbance so that the highest positive peak gets a Y value of +1 [29].

### NMR Experiments

The NMR spectra were collected on an Agilent (Varian) 600 MHz Inova NMR spectrometer (Agilent, Santa Clara, California) with an HCN cold probe. See Materials S1 for details.

### Fluorescence Measurements

Measurements were carried out with a Tecan M1000 (Männedorf Switzerland). 0.2 µM (5′ FAM and 3′ TAMRA) labeled single stranded oligos were incubated in 20 mM Tris pH 8.0 containing different salt concentrations for 10 h. Excitation of fluorophore-tagged oligos were carried out at 475 nm (excitation wavelength of FAM) and emission at 518 nm (emission wavelength of FAM) and 585 nm (emission wavelength of TAMRA) was followed at 25°C, slit widths of 12 nm [30]. The effects of sodium and potassium on the normalized intensity ratio of are presented where zero denotes the ratio value in the absence of salts [30].

## Results

### In vitro and In vivo Binding of Vimentin to Human ALOX15 Promoter Variants

To identify proteins that may bind to the polymorphic ALOX15 promoter, we first performed a DNA-protein pull-down assay. HPLC-purified biotinylated oligomers, 29 base-pairs in length (denoted 29G and 29A), were synthesized according to the human ALOX15 promoter sequence with alternative G and A alleles at rs2255888, respectively. They were then annealed with oligomers of respective complementary sequences to produce double-stranded duplexes (denoted 29G* and 29A*) (Figure 1A). The biotinylated duplex 29G* was incubated with NIH3T3 nuclear extract (lacking the particular human ALOX15 polymorphic promoter sequence) and resulting DNA-protein complexes were analyzed by mass spectrometry (Figure 1B). Based on the detection of multiple peptides, the protein most abundantly bound to the duplex 29G* oligos was determined with high confidence to be vimentin, a cytoskeletal protein (24). Vimentin binding to the ALOX15 promoter region was then tested in vivo. Chromatin immunoprecipitation (CHIP) assay of P1 luciferase-transfected NIH3T3 cells using anti-vimentin (C-20) antibody (raised against the C-terminus of vimentin) was carried out. Figure 1.C shows binding of vimentin to the ALOX15 promoter region under physiological conditions.

### Determination of Promoter Activities of G- and A- Haplotypes in Vimentin-positive and Vimentin-negative Cell Lines

**Figure 2:**
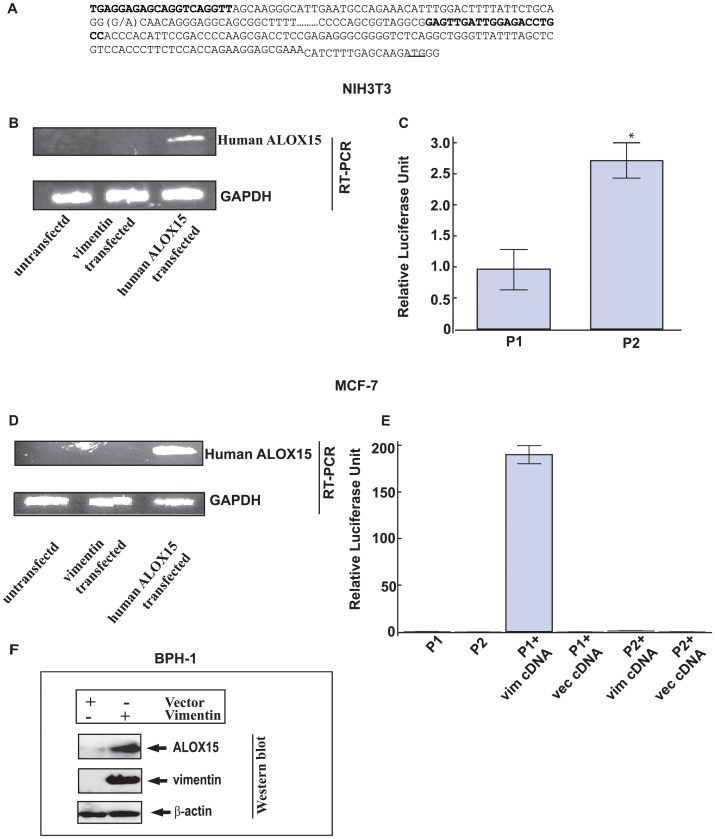
Promoter activities of ALOX15 variants in NIH3T3 cells and MCF-7 cells. Vimentin binds to ALOX15 promoter (**A**) ALOX15 promoter sequence. Primers used for this study and alternative alleles of rs2255888 are shown in bold. (G/A) in bold denotes SNP rs2255888 position. (**B**) Qualitative measurement of human ALOX15 (upper panel) and GAPDH (lower panel) gene expression by RT-PCR in NIH3T3 cells. (**C**) Luciferase activities of P1 and P2 transfected NIH3T3 cells was measured after 24 h. *denotes p = 2×10^−5^ vs. P2. The results are the average of two independent transfections performed in triplicate ±S.E. (**D**) Human ALOX15 (upper panel) and GAPDH (lower panel) gene expression by RT-PCR in MCF-7 cells in presence of vimentin. (**E**) Luciferase activities of P1 and P2 transfected MCF-7 cells were measured after 24 h. The experiments were done in triplicates. **denotes p<0.0001 between the experimental sets. Transfection of NIH3T3 cells and MCF-7 cells with human ALOX15 cDNA for 24 hr was performed separately as control in gene expression experiments. pRL and pTK luciferase constructs were co-transfected in NIH3T3 and MCF-7 respectively. Luciferase activity was normalized using pRL and pTK luciferase activity in NIH3T3 and MCF-7 respectively. The results are the average of three independent transfections performed in triplicate ± S.E. (**F**) Western blot of vector (CMV) and vimentin cDNA transfected BPH-1 cells. The cells were harvested after 48 h. The cells were lysed with lysis buffer and loaded on to the SDS-PAGE Gel. The blot was probed with anti-ALOX15 antibody (1∶2000), anti vimentin antibody (1∶1000) and anti-β-actin antibody (1∶7000).

To characterize the promoter variants, we cloned the ALOX15 promoter regions, from −701 to −108 base pairs upstream from the transcription start site, with either the G (Promoter 1, P1) or the A allele (Promoter 2, P2) of rs2255888 (Figure 2 A). This region has been shown to exhibit transcriptional activity in a previous report [31]. Figure 2 B verifies the absence of human ALOX15 transcript in untransfected and vimentin-transfected NIH3T3 cells. We carried out the transfection of the cloned human luciferase promoter constructs in NIH3T3, a mouse fibroblast cell line. P2 showed a higher promoter activity compared to P1 (p<0.0001) (Figure 2 C). Before characterizing vimentin-mediated promoter activity in MCF-7, a breast cancer cell line lacking vimentin and endogenous ALOX15 protein, we checked the status of ALOX15 expression in this cell line. Endogenous ALOX15 transcript was not detected in MCF-7 cells (Figure 2 D). This is also supported by reports demonstrated reduction of ALOX15 expression in breast cancer [32], suppression of ALOX15 promoter (−120 to −391) activity by NURD complex in colon cancer [31] and a previous report [33]. We carried out transfection of the cloned human luciferase promoter constructs. Luciferase activity was significantly greater in P1 transfected cells in the presence of vimentin as compared to vector transfected cells (p<0.0001) (Figure 2 E). A significant increase of vimentin-mediated P1 promoter activity was noted in NIH3T3, and in vimentin-transfected MCF-7 cells, whereas P2 showed different promoter activities in these cells (Figure 2 C and Figure 2 E). The reason for a difference in the relative luciferase activity of P1 and P2 promoters in presence of vimentin is unknown but may be explained by the presence of other competing proteins in these cells, which may interfere with vimentin binding. ALOX15 protein could not be detected in untransfected and vimentin-transfected MCF-7 suggesting that this cell line may not be a suitable model for these experiments because possible co-factors necessary to the vimentin-mediated ALOX15 regulation may be different in this cell line. Therefore, we repeated the experiments in a BPH-1 cell line, a benign prostate cell line, which expresses vimentin at low level. BPH-1 cells were transfected with vimentin construct and vector (CMV) construct separately. Figure 2 F shows the expression of ALOX15 protein in the vimentin-transfected cells although ALOX15 protein was not in the detectable range in the vector (CMV) transfected cells.

### Specific in vitro Binding of Vimentin to Promoter Variants

**Figure 3:**
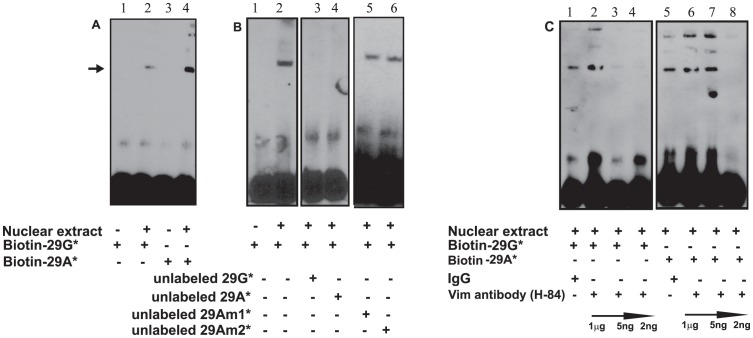
Specific *in vitro* Binding of Vimentin to Promoter Variants. (**A**) Biotin-labeled duplex oligos and 10 µg NIH3T3 nuclear extract were used for the experiments. Arrow indicates protein binding to the oligos. (**B**) Determination of specificity of duplex 29G* by completion with unlabeled duplex oligos. (**C**) Specific binding of vimentin to 29G* and 29A* oligos was demonstrated with anti-vimentin antibody.

*In vitro* binding was further studied with electrophoretic mobility shift assay (EMSA). Biotinylated double-stranded duplex oligos 29G* and 29A* were each incubated with NIH3T3 nuclear extract and electrophoresed on polyacrylamide gel at 4°C. There was a higher nuclear protein binding for 29A* as compared to 29G* (Figure 3 A). The specificity of binding of the nuclear protein(s) to the oligos 29G was verified in presence of 100-fold excess unlabeled duplex oligos. Oligo 29A* competed with 29G* (Figure 3 B lane 4) whereas duplexes containing multiple nucleotide substitutions (Am1* and Am2*, see in supplemental Table S1) did not show any competition with 29G* (Figure 3 B, lane 5 and lane 6). To confirm the binding of vimentin to the oligos, we preincubated anti-vimentin antibody (H-84, raised against the N-terminal peptide region of vimentin, which includes a DNA binding motif) with the oligos in EMSA. Reduction in band intensity observed with the incubation of different amounts of anti-vimentin antibodies (1 µg, 1∶200 and 1∶1000) (Figure 3 C, lanes 3, 4, 8) indicated a competitive binding between duplex and anti-vimentin antibody to the N-terminal region of vimentin. Competition was seen with lower dilution of the antibody which may be explained by the fact that the antibody in general binds its ligand at a particular concentration.

Previous reports of a direct physical interaction of vimentin to telomeric DNA sequence [34] and the known DNA binding motif in the N-terminus of vimentin [35] raise the possibility of a direct physical binding of vimentin to the polymorphic promoter sequence. Presence of low mobility bands may be due to oligos interacting with multimeric forms of vimentin [36], [37], [38]. We found that labeling of the oligos with the biotin did not hinder the binding of vimentin to the oligos.

### Higher-Order Structure in Duplex and Single Stranded Oligonucleotides

**Figure 4:**
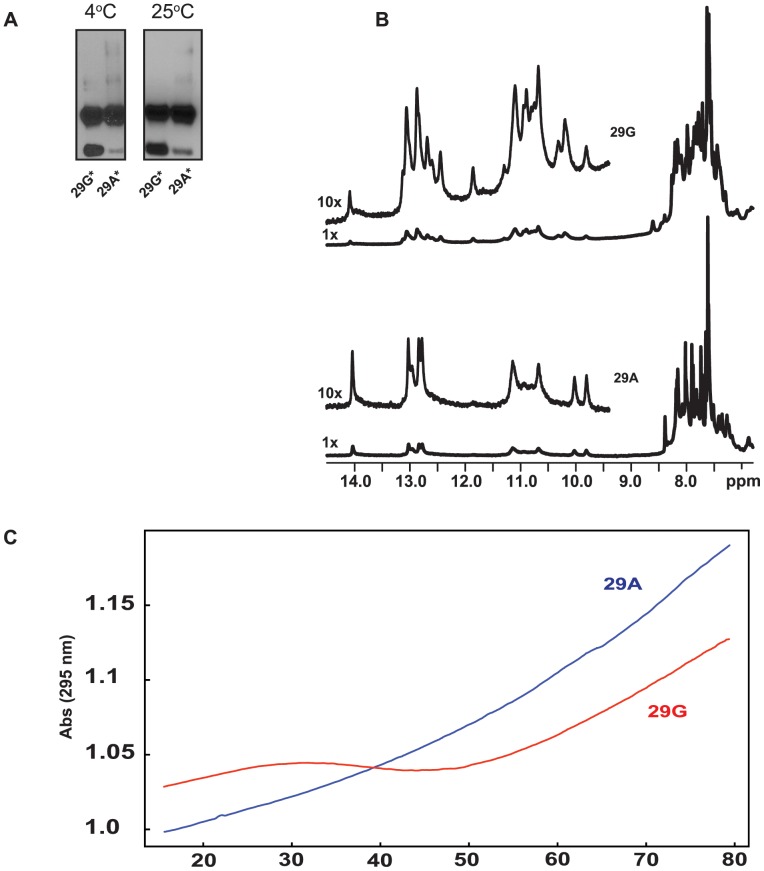
Higher-Order Structure in Duplex and Single Stranded Oligonucleotides. (**A**) 15% non-denaturing polyacrylamide gel electrophoresis of duplex oligos in the presence of 40 mM NaCl. The oligos were incubated in binding buffer (Materials and Methods) for 40 min and 20 min at 4°C and 25°C respectively. 29G* (Lanes1 and 3), 29A* (Lanes 2 and 4) are at 4°C and 25°C respectively. (**B**) 1D proton NMR spectra (imino, amino and aromatic signal regions) of oligonucleotides 29G (top) and 29A (bottom) in the presence of 40 mM Na+, 10 mM phosphate, pH 7.0. (**C**) Denaturation profiles of single stranded oligos 29G (red) and 29A (blue) at 295 nm wavelength. Experimental conditions: 40 mM Na+, 10 mM phosphate, pH 7.0.

We next investigated possible differences in higher order structure between the two double-stranded duplexes by non-denaturing gel analysis. Biotinylated oligos 29G* and 29A* were each incubated in the same high-salt buffer as that used in the in vitro binding experiments for either 40 min. or 20 min., at either 4°C or at 25°C. They were then electrophoresed through a 15% non-denaturing polyacrylamide gel in the presence of 40 mM NaCl. Electrophoretic patterns were similar for both duplexes but a band of higher mobility showed a reduced intensity for 29A* as compared to 29G* (Figure 4 A). This high-mobility band corresponds to the single stranded oligo as shown in Figure S1A. We verified that the presence of the single strand in the experiment was not due to the improper or incomplete annealing of the complementary oligos. Changing the stoichiometry of the oligos during the annealing process did not affect the intensity of the high-mobility band (Figure S1 B). Low mobility bands in Figure 4 A were also detected in the electrophoretic pattern of single stranded oligos 29G, 29A, and the complementary strand of 29A but not the complementary strand of 29G (Figure S1A). These differences in electrophoretic patterns may be due to differences in higher- order DNA conformation of the promoter haplotypes.

One-dimensional proton NMR studies were also carried out with 29G and 29A (Figure 4 B). The imino proton spectra observed for 29G (upper panel) and 29A (lower panel) were significantly different. The sharp, intense imino signals observed for 29G in the 12–14 p.p.m range indicates that 29G is forming a well-defined folded structure compared to 29A. Fewer peaks in the 1D proton spectrum of the 29A oligo at 10–12 p.p.m compared to the 29G oligo also indicated that 29A is likely less structured than 29G (Figure 4 B).

To probe the thermodynamics of the two single stranded oligos, 29G and 29A, we utilized ultraviolet melting (UVM) experiments by monitoring the UV absorbance at 260 nm and 295 nm [28] during thermal denaturation (15–80°C) and renaturation (80–15 C). The 29G and 29A sequences showed distinctively different transition profiles at 295 nm (Figure 4 C). Visually, the profile for 29G showed a single unfolding transition across a broad temperature range of 30°C with a melting temperature (Tm) of 45.1±1.0°C and 29A did not display a transition over the temperature range examined. The Gibbs free energy for the 29G oligonucleotide at 25°C and 37°C was calculated to be −9.2±1.0 and −3.7±0.6 kJ•mol^−1^•K^−1^, respectively. These values indicate a weakly stabilized secondary structure in the 29G single stranded oligo under these experimental conditions and repeated measurements showed this folding/unfolding transition to be completely reversible. From the slope of van’t Hoff graph and y-intercept, the enthalpy and entropy were calculated as −35.2±2.7 kcal•mol^−1^•K^−1^ and −110.6±8.2 cal•mol^−1^•K^−1^ (Figure S2).

### Salt Induced Folding of Single Strand Promoter Variant Oligos

**Figure 5:**
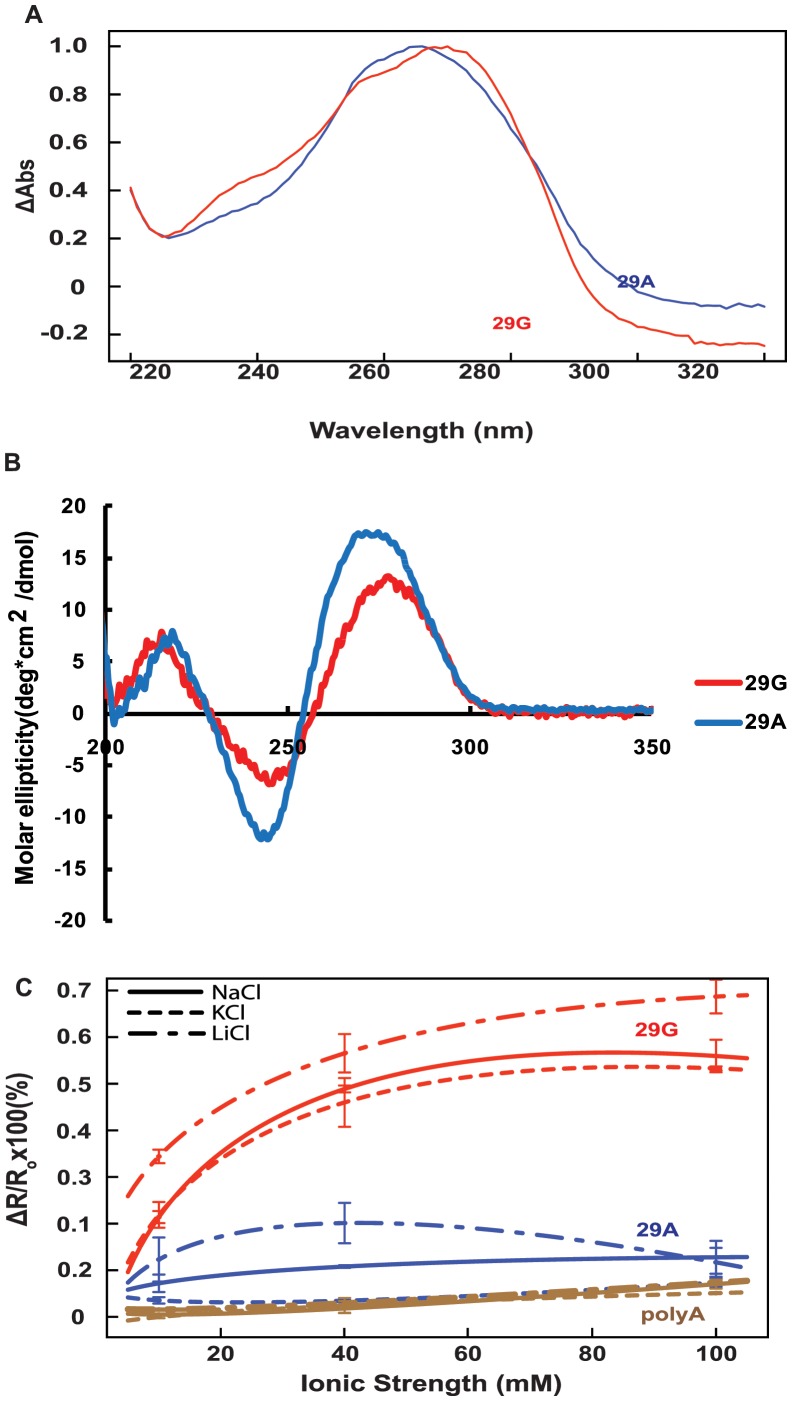
Salt Induced Folding of Single Strand Promoter Variant Oligos. (**A**) Normalized differential absorbance spectra of 29G (red) and 29A (blue). (**B**) CD spectra of 29G and 29A in presence of 40 mM NaCl. (C) Relative fluorescence ratio values in the presence of monovalent cations (Na+, K+, Li+) for 29G (red) and 29A (blue). *R* = *F*em, 585/*F*em, 518 calculated from emission spectra, *λ*ex = 475 nm. PolyA (brown) used as a non-structured control.

We carried out thermal difference spectra (TDS) by scanning wavelength from 220 nm to 320 nm at different temperatures (from 20°C to 80°C) to detect the stability of the conformational forms for each of the oligos. Scanning of 29G and 29A showed different thermal scanning profiles which indicates a difference in the stability of the oligos as shown in Figure 5 A.

Next, we analyzed the folding pattern of the single stranded oligos by circular dichroism (CD). The profiles for both the oligos showed the presence of higher order structure. However, non-overlapping spectral profiles indicate a difference in the folding pattern of the two oligos as shown in Figure 5 B.

Förster resonance energy transfer (FRET) studies were carried out with double-labeled single-stranded oligos under various NaCl, KCl, and LiCl concentrations, including high Na+ concentration and high K+ concentration. The FRET efficiency, measured by the ratio of fluorescence at 518 nm and 588 nm, was higher for 29G than 29A, indicating a change in folding pattern with increasing salt concentrations (Figure 5 C).

## Discussion

ALOX15, a member of lipoxygenase family, has been implicated in the pathogenesis of cardiovascular disease through its effects on atherogenesis, vascular function and remodeling [39]. Data from *in vitro* study and from animal models suggest both an atheroprotective and a pro-atherogenic role of ALOX15 [4], [8]. A better understanding of the mechanisms of regulation of this gene may provide important clues about its role in health and disease. Sequence variation in the ALOX15 gene has been previously identified and associated with coronary artery disease but the biological mechanisms underlying these associations are unexplored [5], [7], [9]. The present study sought to characterize the functional impact on ALOX15 gene regulation of rs2255888, a promoter polymorphism previously associated with carotid plaque burden [10]. To the best of our knowledge, this is the first report to show a vimentin interaction with the ALOX15 promoter. Here, we have presented evidence supporting that vimentin, a cytoskeletal protein, regulate ALOX15 promoter activity. We have identified a region in the ALOX15 promoter with a higher-order DNA structure, which may be a site for vimentin-mediated regulation of ALOX15 promoter activity.

We selected two cell lines that do not express the human ALOX15 gene, including a vimentin-expressing mouse fibroblast cell line (NIH3T3) and a human breast cancer cell line (MCF-7), a vimentin-lacking cell lines. We characterized the human ALOX15 G-carrying promoter haplotype, thus, our study cannot rule out the specificity of vimentin binding to this promoter sequence only. The CHIP assay showed specificity of *in vivo* vimentin-binding to G-carrying promoter haplotype as determined by pulling DNA-vimentin complex with anti-vimentin antibody only but not with IgG. To further investigate vimentin-mediated promoter activity, we carried out transfection experiments and promoter assay in MCF-7 cells that lack endogenous vimentin and ALOX15. Significant promoter activity of P1 luciferase in vimentin cDNA-transfected MCF-7 cell compared to vector cDNA-transfected cells was also observed in these cells. The reason for a difference in the relative luciferase activity of P2 promoter in NIH3T3 and in vimentin-transfected MCF-7 cells is unknown and needs further study. There was no endogenous ALOX15 expression in vimentin-transfected NIH3T3 and MCF-7 cells and this was not due to unsuccessful transfection as indicated by control experiments. Whether vimentin directly binds the ALOX15 promoter or indirectly through other interacting proteins is unclear. A recent study has shown the ability of vimentin to regulate osteocalcin transcription in immature osteoblasts through interaction with a transcription factor (ATF4) [21]. Differential distribution of such an interacting molecule(s) between NIH3T3 and MCF-7 cell lines may explain differences in P2 promoter activity between the two cell lines. We also investigated vimentin-mediated ALOX15 expression by transfection of vimentin cDNA in BPH-1, a benign prostate cell line that expresses vimentin at a low level. We showed that vimentin regulated ALOX15 expression at the protein level, further strengthening our suggestion of a role of vimentin as a regulator of ALOX15.

Whether the effect of vimentin on ALOX15 promoter activity has an impact on atherosclerosis remain to be determined. Nonetheless, our findings add to the growing body of evidence that has suggested a role of vimentin in cardiovascular disease. Indeed, vimentin, a highly abundant protein in human monocytes and activated macrophages [12], [13] has been reported to be upregulated by LDL [16]. An evidence of specific binding of vimentin to oxidized LDL has been demonstrated [40]. A report also demonstrated high expression of vimentin in growth factor exposed smooth muscle cells which are involved in atherosclerosis [41].

We propose that sequence variation in the ALOX15 promoter may result in variation in higher-order structure of the promoter. The ALOX15 promoter variant rs2255888 exhibited a difference in promoter activity, DNA conformation, and vimentin-binding ability. Vimentin has been shown to bind to a canonical G-quadruplex structure [24]. Here we have showed vimentin-binding to a higher order structure, which is not a canonical G-quadruplex. Whether vimentin-binding is direct or indirect through interaction with other factors remains to be explored. Our data also suggest vimentin mediated ALOX15 protein expression. Further studies will determine whether a new role of vimentin in ALOX15 regulation and the sequence variation at rs2255888 in the ALOX15 is relevant to ALOX15 implication in the pathogenesis of atherosclerosis.

## Supporting Information

Figure S1 **Verification of presence of higher order structure in single and double stranded oligos.** The oligos were incubated in binding buffer used in EMSA which for 20 min at 25°C. **(A)**. 29G* 1∶1 ratio (Lane1), 29A* 1∶1 ratio (Lane 2), 29G (Lane 3), complementary strand of 29G (Lane 4) 29A (Lane 5), complementary strand of 29A (Lane 6). **(B)**. Determination of proper annealing of single stranded oligos with its complementary strands. Different proportions of oligos were annealed. Lane1–29G* where 29G and * are (0.95∶1); lane2–29G* (0.9∶1); lane3–29G*(1∶0.95); lane4–229G*(1∶0.9); lane5–29A*(0.95∶1); lane6–29A*(0.90∶1); lane7–29A*(1∶0.95); lane8–29A*(1∶0.9). 15% nondenaturing polyacrylamide gel was run in presence of 40 mM NaCl for both the experiments.
(TIF)

Figure S2
**TOP: Van’t Hoff plot. LnKa plotted as a function of the inverse of temperature (K^−1^).** The linearity of this plot validates the two-state model used to describe the folded-unfolded transition. From the slope of this graph and y-intercept, the van’t Hoff enthalpy and entropy were calculated as −35.2±2.7 kcal•mol^−1^•K^−1^ and −110.6±8.2 cal•mol^−1^•K^−1^, respectively. From the standard thermodynamic relation 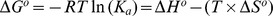 the free energy at 25°C and 37°C was calculated to be −9.2±1.0 and −3.7±0.6 kJ•mol^−1^•K^−1^, respectively. **Bottom:** Theta folded plot representing the fraction of folded 29GG as a function of temperature, determined after transposing the raw data to upper and lower baselines as described in materials and methods. The melting temperature (Tm = 45.1+/−1.0°C) in 40 mM Na^+^,10 mM phosphate, pH 7.0 was defined as temperature at which half the oligo is folded (θ = 0.5). Highlighted in red are values in which (0.15 < θ < 0.85), where the Ka is most accurately known and is the region used for generating the van’t Hoff plot and extracting thermodynamic parameters.(TIF)

Table S1 **Oligos used for making duplex oligos and for competition experiment in EMSA.** Single stranded oligo 29G was further modified with additional nucleotide substitutions and denoted as 29Am1 and 29Am2. The 29Am1* and 29Am2* duplexes were made with 29Am1 and 29Am2 with the respective complementary strands.
(TIF)

Materials S1 **Supporting Materials**
(DOC)

Methods S1 **Supporting Methods**
(DOCX)
