# Supplementary figures and images for: Characterization of a Human 12/15-Lipoxygenase Promoter Variant Associated with Atherosclerosis Identifies Vimentin as a Promoter Binding Protein
Source: PLoS One. 2012 Aug 7;7(8):e42417. doi: 10.1371/journal.pone.0042417 (PMC3413658; doi:10.1371/journal.pone.0042417)

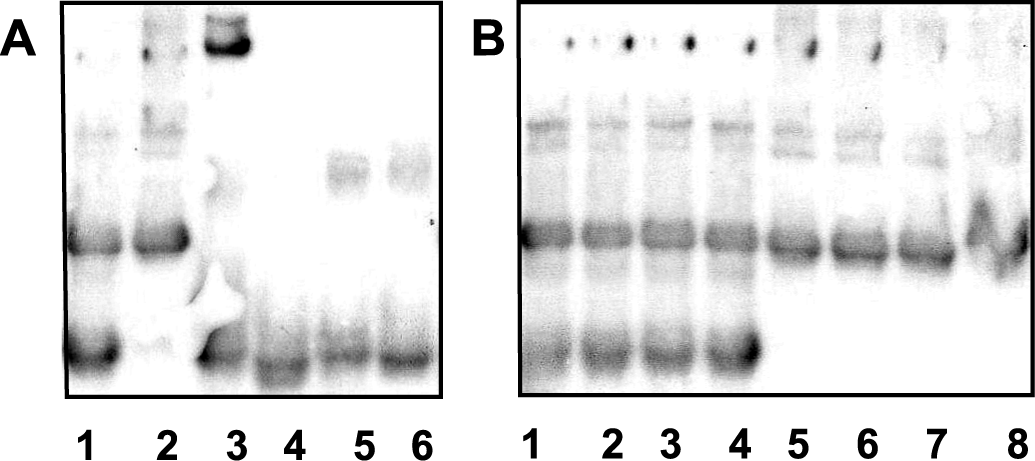

Supplement: Figure S1 — Verification of presence of higher order structure in single and double stranded oligos. The oligos were incubated in binding buffer used in EMSA which for 20 min at 25°C. (A). 29G* 1∶1 ratio (Lane1), 29A* 1∶1 ratio (Lane 2), 29G (Lane 3), complementary strand of 29G (Lane 4) 29A (Lane 5), complementary strand of 29A (Lane 6). (B). Determination of proper annealing of single stranded oligos with its complementary strands. Different proportions of oligos were annealed. Lane1–29G* where 29G and * are (0.95∶1); lane2–29G* (0.9∶1); lane3–29G*(1∶0.95); lane4–229G*(1∶0.9); lane5–29A*(0.95∶1); lane6–29A*(0.90∶1); lane7–29A*(1∶0.95); lane8–29A*(1∶0.9). 15% nondenaturing polyacrylamide gel was run in presence of 40 mM NaCl for both the experiments. (TIF) [file pone.0042417.s001.tif]

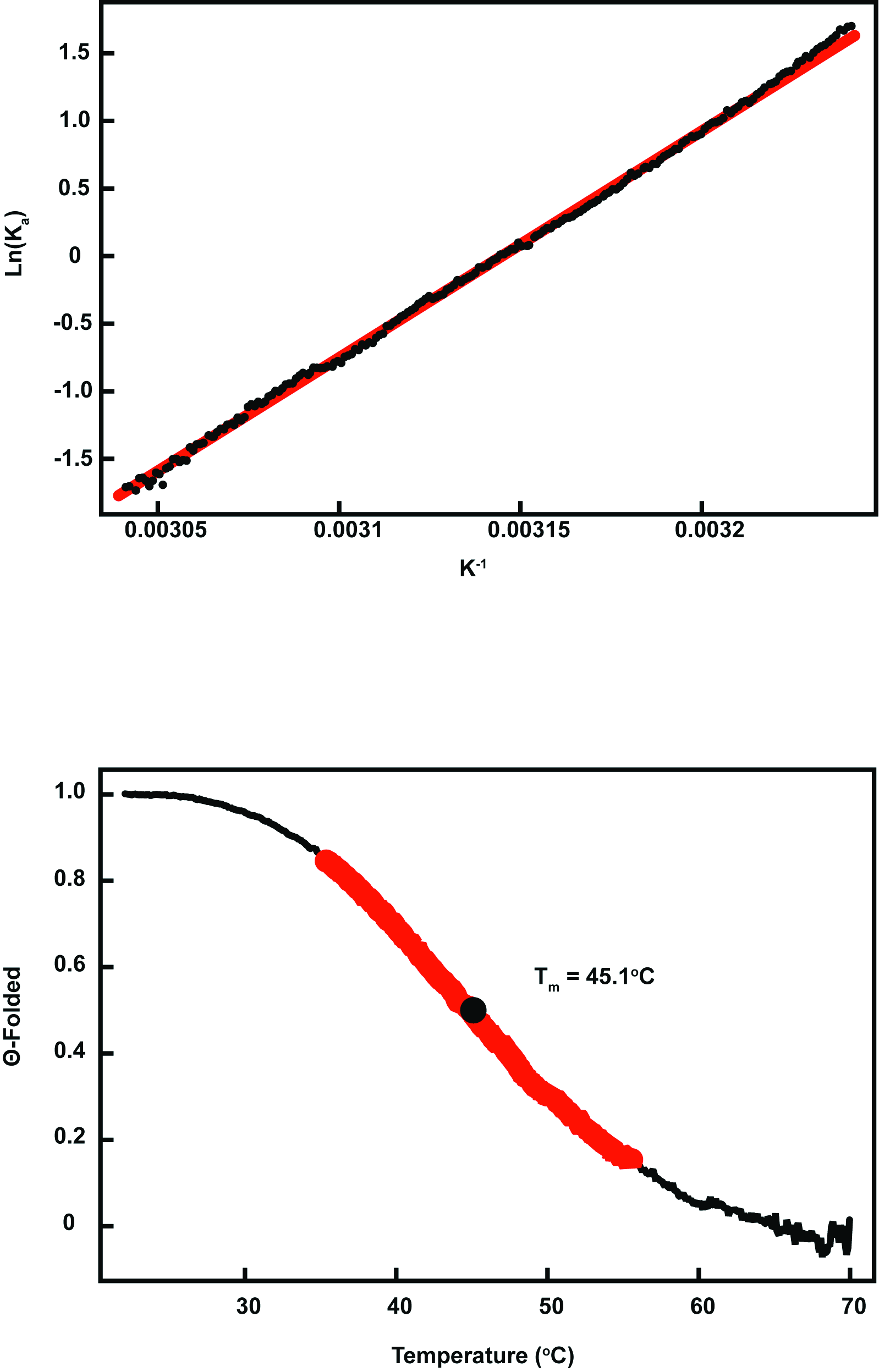

Supplement: Figure S2 — TOP: Van’t Hoff plot. LnKa plotted as a function of the inverse of temperature (K−1). The linearity of this plot validates the two-state model used to describe the folded-unfolded transition. From the slope of this graph and y-intercept, the van’t Hoff enthalpy and entropy were calculated as −35.2±2.7 kcal•mol−1•K−1 and −110.6±8.2 cal•mol−1•K−1, respectively. From the standard thermodynamic relation the free energy at 25°C and 37°C was calculated to be −9.2±1.0 and −3.7±0.6 kJ•mol−1•K−1, respectively. Bottom: Theta folded plot representing the fraction of folded 29GG as a function of temperature, determined after transposing the raw data to upper and lower baselines as described in materials and methods. The melting temperature (Tm = 45.1+/−1.0°C) in 40 mM Na+,10 mM phosphate, pH 7.0 was defined as temperature at which half the oligo is folded (θ = 0.5). Highlighted in red are values in which (0.15 < θ < 0.85), where the Ka is most accurately known and is the region used for generating the van’t Hoff plot and extracting thermodynamic parameters. (TIF) [file pone.0042417.s002.tif]

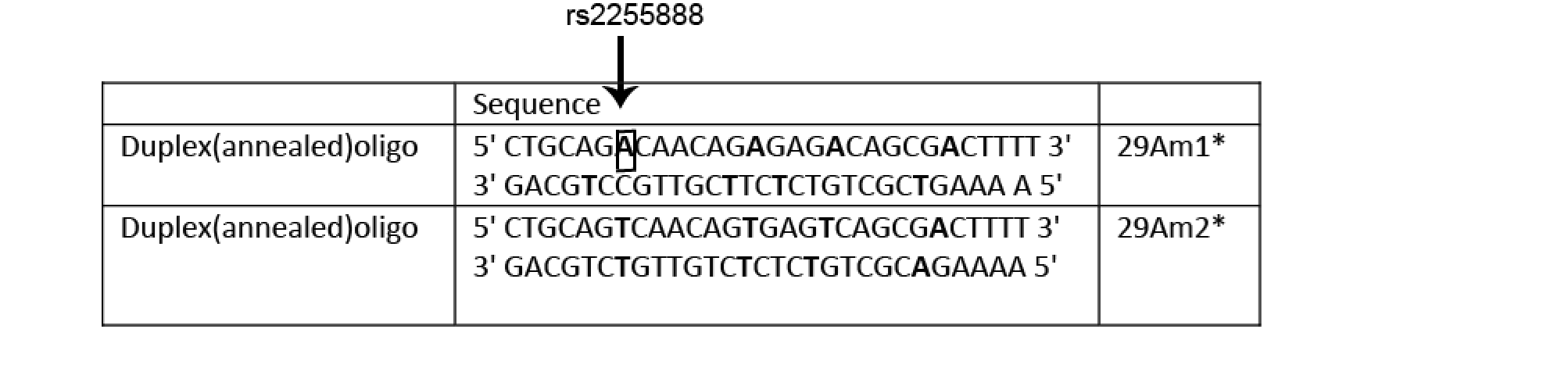

Supplement: Table S1 — Oligos used for making duplex oligos and for competition experiment in EMSA. Single stranded oligo 29G was further modified with additional nucleotide substitutions and denoted as 29Am1 and 29Am2. The 29Am1* and 29Am2* duplexes were made with 29Am1 and 29Am2 with the respective complementary strands. (TIF) [file pone.0042417.s003.tif]
