# Supplementary material for: Characterization of a Human 12/15-Lipoxygenase Promoter Variant Associated with Atherosclerosis Identifies Vimentin as a Promoter Binding Protein
Source: PLoS One. 2012 Aug 7;7(8):e42417. doi: 10.1371/journal.pone.0042417 (PMC3413658; doi:10.1371/journal.pone.0042417)
Supplement: Materials S1 — Supporting Materials (DOC) [file pone.0042417.s004.doc]

**Supporting Information**

Characterization of a Human 12/15-Lipoxygenase Promoter Variant Associated with Atherosclerosis Identifies Vimentin as a Promoter Binding Protein *

**Susmita Samanta^1^, Kurtis Anderson^2,4^, Sean Moran^5^, David Hawke^6^, David Gorenstein^2^, MyriamFornage^1,3^**

^1^Research Center for Human Genetics, Brown Foundation Institute of Molecular Medicine, University

of Texas Health Science Center at Houston, Houston, Texas, United States of America,

^2^Centers for Proteomics and Systems Biology, Brown Foundation Institute of Molecular Medicine,

University of Texas Health Science Center at Houston, Houston, Texas, United States of America,

^3^Human Genetics Center, School of Public Health, University of Texas Health Science Center at

Houston, Houston, Texas, United States of America,

^4^Department of Biochemistry and Molecular Biology, University of Texas Medical Branch, Galveston,

Texas, United States of America,

^5^Department of Biochemistry & Cell Biology, Rice University, Houston, Texas, United States of America,

^6^Department of Molecular Pathology, M.D. Anderson Cancer Center, Houston, Texs, United States of America

Current address of Susmita Samanta: Baylor college of Medicine, Houston, Texas, United States of America. Email: [Susmita.Samanta@bcm.edu](mailto:Susmita.Samanta@bcm.edu). Present address of Sean Moran Uniformed Services University of the Health Sciences, Bethesda, Maryland, United States of America

**This PDF file includes:**

Materials and Methods

Figures S1 and S2

Table S1

**Materials**

Fire fly (pGL4.10) and renilla luciferase (pGL4.74 hRluc/TK) vectors were purchased from Promega (St. Louis, MO). Mouse fibroblast cell lines (NIH 3T3) was purchased from American Tissue Culture Type (ATCC) and maintained in DMEM with 10% FBS. MCF-7 was cultured in DMEM-F12 medium with 10% FBS. Fugene 6 (Roche Bioscience, Indianapolis, IN) was used for transfection. BPH-1 cell line is a gift from Dr. Hayward Simon, Vanderbuilt University Medical Center. Site-Directed mutagenesis was done with QuikChange II kit from Stratagene) NE-PER nuclear and cytoplasmic extraction reagent, Halt Protease and phosphatase inhibitors, chemiluminescent nucleic acid detection module were purchased from Thermo Scientific. ChIP-IT™ Express Enzymatic Kit (Active Motif, Carlsbad, CA) was used for CHIP assay. HPLC purified biotinylated fluorescence labeled and unlabeled oligonucleotides were purchased from Integrated DNA technology, (IDT, Coraville, IA). Oligonucleotides purified by HPLC (Midland Certified Reagent Company) were used for UVM and NMR studies. Anti vimentin antibody for DNA protein pull down assay (H-84 and C-20) were purchased from Santa Cruz Biotechnology (Santa Cruz, CA) and for western blot from Cell signaling (Boston, MA). pCMV-Tag2 was purchased from Stratagene. GFP tagged ALOX15, vimentin cDNA was purchased from Origene (Rockville, MD). Anti ALOX15 antibody were purchased from Origene (Rockville, MD). β-actin was purchased from Sigma ( St. Louis, MO). Trizol used for RNA isolation was purchased from Invitrogen, Carlsbad, CA. cDNA Reverse Transcription Kit (Life Technologies, Carlsbad, CA) was used for cDNA synthesis. Specific ALOX15 and GAPDH primers set for real time PCR were purchased from Applied Biosystems, Carlsbad, CA.
