# Supplementary material for: Characterization of a Human 12/15-Lipoxygenase Promoter Variant Associated with Atherosclerosis Identifies Vimentin as a Promoter Binding Protein
Source: PLoS One. 2012 Aug 7;7(8):e42417. doi: 10.1371/journal.pone.0042417 (PMC3413658; doi:10.1371/journal.pone.0042417)
Supplement: Methods S1 — Supporting Methods (DOCX) [file pone.0042417.s005.docx]

**Methods**

**Cloning of the ALOX15 Promoters and cDNA**

594 bp promoter sequence of the ALOX15 gene of (-701 to -108 upstream from the translational start codon) was PCR amplified from human genomic DNA of normal subjects (written consent was obtained for the subjects included in this research approved by the Institutional Review Board of the University of Texas Health Science Center at Houston) using the forward primer 5’ CCCTCGAGTGAGGAGAGCAGGTCAGGTT 3’ and the reverse primer 5’ AAGCTTGGCAGGTCTCCAATCAACT 3’. The PCR products were sequenced to check for the correct sequence. PCR amplified ALOX15 promoter fragment was cloned into a pGL4.10 (luc2) vector. The promoter constructs were verified by sequencing analysis. Site directed mutagenesis was done to change nucleotide G to A. Two constructs carrying G haplotype and A haplotype sequences were made. ALOX15 was cloned in pCMV-Tag2 using forward and reverse primer having BamH1 and Xho1 sequence respectively. The study was.

**Annealing of Oligos**

The HPLC purified biotinylated oligos were annealed with their respective complementary strands to make duplex oligos in a buffer containing 10 mM Tris-Hcl pH8.0, 50 mM NaCl and 1 mM EDTA at 95^o^C for 12 min, 65^o^C for 5 min, 55^o^C for 5min, 37^o^C for 5min, 25^o^C for 5 min.

**DNA Pull Down Assay**

50 μl beads was washed with 2x B&W buffer twice (10 mM Tris,1 mM EDTA, 2.0 M NaCl, pH 7.9). Incubation of biotinylated 29G* annealed oligos (20 pM) with beads was carried out in 1x B&W buffer for 15 min at room temperature with gentle shaking to prevent precipitation in solution. The beads were washed two times with 1x B&W buffer and three times with incubation buffer (50 mM Tris, 1mM EDTA,100 mM KCl, adjust pH 7.9, 5% Glycerol, 0.1% TritonX-100, 1mM DTT. Beads bound oligos were incubated with 5µg/ml of cell extract (100 µl) for 30 min at room temperature with gentle shaking. The washed beads were used for mass spectrometry using LC/MS/MS mass spectrometer (Thermo Fisher Scientific, Waltham, MA**)** to identify the bound proteins. Elution of the protein was done with TFA in ACN-H_2_O following ABI’s protocol. All MS/ MS samples were analyzed using Mascot (Matrix Science; version Mascot).

**Western Blot**

Lysates from cell lines were made by cell lysis buffer (Cell signaling) containing protease and phosphatase inhibitors. The lysates were passed through 22-G needle attached to a 1-ml syringe and centrifuged. The supernatant was separated and Protein concentration of the supernatant was then determined using the BCA Protein Assay (Rockford, IL). 50 µg protein lysate were denatured by the addition of 5X sample loading buffer (National Diognostics, Charlotte, NC) and kept for 5 mins at 100^o^C. The lysate was electrophoresed on a 4–20% Tris-Bis polyacrylamide (Bio-Rad, Hercules,CA). Proteins were then transferred to Immobilon-P (Millipore, Bedford, MA) using the Biorad semi dry transfer apparatus (Hercules, CA). The membrane was subsequently blocked with 5% nonfat dry milk in Tris-buffered saline containing 0.05% Tween 20. The immunoblot was probed with with anti-ALOX15 antibody (1:2000), anti vimentin antibody (1:1000) and anti-β-actin antibody (1: 7000) to detect ALOX15, vimentin and β-actin. Detection was performed using the Super Signal West Pico Chemiluminescent Substrate kit (Pierce, Rockford, IL) for β-actin, ECL Plus western blotting detection system (GE Health Care. Piscataway, NJ) was used for ALOX15 and vimentin detection.

**Mass spectroscopy Analysis**

Two fractions were taken from the beads: a water-eluted fraction, after which the beads were eluted with 2% TFA in 30% acetonitrile-water. The eluted fraction was acetone precipitated (-20°C, overnight), then the precipitate was resuspended in 30 mM NH_4_HCO_3_ and digested with 100 ng of modified trypsin (sequencing grade, Promega, Madison, WI)at 37°C overnight. The resultant peptides were analyzed by nano-liquid chromatography-coupled ion trap mass spectrometry with on-line desalting on a system consisting of a FAMOS autosampler, an Ultimate Nano liquid chromatography module, and a Switchos pre-column switching device on a 75-µm × 150-mm C-18 column (all from Dionex Corp., Sunnyvale, CA). Electrospray ion trap mass spectrometry was performed on an LTQ linear ion-trap mass spectrometer (Thermo Fisher Scientific, Waltham, MA). Proteins were identified by database search of the fragment spectra against the National Center for Biotechnology Information nonredundant protein database (NCBInr) using Mascot (version 2.2, Matrix Science, London, UK). The sequence match for one of the assigned peptides is shown with the fragment ions identified as y(n), b(m) to indicate the y and b ions, respectively. The table shows the top-ranked peptide match and the scores for the next 9 for that spectrum.

**Chromatin Immunoprecipitation (CHIP) Assay**

Nuclear proteins were cross-linked to Pl luciferase construct by adding formaldehyde to P1 luciferase construct transfected NIH3T3 to a final concentration of 1% and incubating for 10 minutes at room temperature. After washing and collecting the cells as a pellet, the cells were sheared enzymatically according to manufacturer’s protocol. Magnetic beads coupled with protein G and anti-vimentin antibody C-20 (1µg) were used to capture chromatin immunoprecipitate. Goat IgG (1 µg) was used as a control to determine the specificity of CHIP assay.The precipitate was eluted with 50 μl elution buffer (Active Motif). The eluate was heated to reverse formaldehyde cross-linking which was followed by proteanase K digestion at 37°C for 1 hour. 5 μl of the eluted DNA was used for PCR amplification with promoter-specific primers. PCR product was subjected to electrophoresis on 1.5% agarose gels, stained with ethidium bromide.

**Electrophoretic Mobility Shift Assay**- HPLC purified biotinylated oligos were annealed with their respective complementary strands to make duplex oligos in a buffer containing 10 mM Tris-HCl pH 8.0, 50 mM NaCl and 1 mM EDTA at 95^o^C for 12 min, 65^o^C for 5 min, 55^o^C for 5 min, 37^o^C for 5min, 25^o^C for 5 min. Purity of duplex was checked using non-denaturing polyacrylamide gel electrophoresis. Annealed oligos was incubated with nuclear extracts (10 µg). 6% non-denaturing polyacrylamide was run and blotted in biodyne nylon membrane. The membrane was UV cross-linked and developed using chemiluminescent nucleic acid detection module (Thermo Scientific) according to manufacturer’s protocol.

**Ultraviolet Thermal Melting Studies (UVM)**

Measurements of buffer alone showed no evidence of a thermal dependence of A_295_ and buffer subtraction as well as data smoothing operations was omitted.

**Thermodynamic Analysis of UVM**

Data was collected over four melting cycles and for each ramp the upper- and lower-sloping baselines were determined manually from the graph of absorbance vs. temperature and raw absorbance values were subtracted from these baselines to calculate the fraction of folded oligomer, Θ*_T_*, according to$\Theta_{T}=[A_{295}(T)-A_{U}(T)]/[A_{F}(T)-A_{U}(T)]$. A numerical approximate function was interpolated to this curve and the melting transition of each heating/cooling ramp was determined as its solution where Θ*_T_* =0.5. UVM is a model-dependent approach for obtaining thermodynamic information and the Θ observable (supporting information Figure S2 bottom) was linked to an association constant describing an intramolecular two-state equilibrium between fully associated (folded) and fully dissociated (unfolded) oligonucleotide through:$K_{a}=\Theta_{T}/(1-\Theta_{T})$, as described ([*24*](#_ENREF_24)) and was used for calculating free energy at various temperatures through $\Delta G=-RT\ln(K_{a})$. The van’t Hoff equation can be derived: $\ln(K_{a})=[(-\Delta H^{o}/R)\times(1/T)]+(\Delta S^{o}/R)$ and plotting lnK_a_ vs T^-1^ reveals (supporting information Figure S2Top) a straight line that can be linearly fit to validate the particular model used for describing the association constant as well as choice of baselines. Once fit the van’t Hoff enthalpy (assuming temperature independence) is determined from the slope, $\Delta H^{o}_{vH}=-R\times d\ln(K_{a})/d(T^{-1})$ and the entropic factor determined from the y-axis intercept, ΔS^o^/R.

**NMR experiment**

The NMR spectra were collected on an Agilent (Varian) 600 MHz Inova NMR  spectrometer with an HCN cold probe.  A sweep width of 22.5 ppm in the proton spectrum was used, and 2048 scans were collected at 2^o^C with an acquisition time of 1 sec and a delay between scans of 1sec. Spectra were erenced to the largest peak in the glycerol methylene multiplet at 3.65 ppm (trace amount of glycerol was present in the samples from the Centricon (ultracel -3K membrane, Millipore) spin filter used for buffer exchange); the chemical shift of this peak was determined with respect to trimethylsilylpropionate ([*36*](#_ENREF_36)) peak in a prepared external standard (TSP and glycerol) at 2^o^C. Solvent suppression was  achieved by using a modified Watergate pulse sequence ([*40*](#_ENREF_40)).

**Figure S1. Verification of presence of higher order structure in single and double stranded oligos**. The oligos were incubated in binding buffer used in EMSA which for 20 min at 25^o^C. **(A).** 29G* 1:1 ratio (Lane1), 29A* 1:1 ratio (Lane 2), 29G (Lane 3), complementary strand of 29G (Lane 4) 29A (Lane 5), complementary strand of 29A (Lane 6). (**B)**. Determination of proper annealing of single stranded oligos with its complementary strands. Different proportions of oligos were annealed. Lane1- 29G* where 29G and * are (0.95:1); lane2- 29G* (0.9:1); lane3- 29G*(1:0.95); lane4- 229G*(1:0.9); lane5- 29A*(0.95:1); lane6-29A*(0.90:1); lane7- 29A*(1:0.95); lane8- 29A*(1:0.9). 15% nondenaturing polyacrylamide gel was run in presence of 40 mM NaCl for both the experiments.

**Figure S2. TOP**: **Van’t Hoff plot. LnKa plotted as a function of the inverse of temperature (K^-1^)**. The linearity of this plot validates the two-state model used to describe the folded-unfolded transition. From the slope of this graph and y-intercept, the van’t Hoff enthalpy and entropy were calculated as -35.2 ± 2.7 kcal•mol^-1^•K^-1^ and -110.6 ± 8.2 cal•mol^-1^•K^-1^, respectively. From the standard thermodynamic relation $\Delta G^{o}=-RT\ln(K_{a})=\Delta H^{o}-(T\times\Delta S^{o})$ the free energy at 25^o^C and 37^o^C was calculated to be -9.2 ± 1.0 and -3.7 ± 0.6 kJ•mol^-1^•K^-1^, respectively. **Bottom:** Theta folded plot representing the fraction of folded 29GG as a function of temperature, determined after transposing the raw data to upper and lower baselines as described in materials and methods. The melting temperature (Tm = 45.1 +/- 1.0^o^C)

in 40 mM Na^+^,10 mM phosphate, pH 7.0 was defined as temperature at which half the oligo is folded (θ = 0.5). Highlighted in red are values in which (0.15 < θ < 0.85), where the Ka is most accurately known and is the region used for generating the van’t Hoff plot and extracting thermodynamic parameters.

**Table S1** **Oligos used for making duplex oligos and for competition experiment in EMSA**. Single stranded oligo 29G was further modified with additional nucleotide substitutions and denoted as 29Am1 and 29Am2. The 29Am1* and 29Am2* duplexes were made with 29Am1 and 29Am2 with the respective complementary strands.
